# Elucidation of the Pathogenicity-Associated Regulatory Network in Xanthomonas oryzae pv. oryzae

**DOI:** 10.1128/mSystems.00789-20

**Published:** 2021-03-09

**Authors:** Dehong Zheng, Huihui Wang, Hao Zhong, Wenli Ke, Huifeng Hu, Ming Sun, Lifang Ruan

**Affiliations:** a State Key Laboratory of Agricultural Microbiology, College of Life Science and Technology, Huazhong Agricultural University, Wuhan, People’s Republic of China; b State Key Laboratory for Conservation and Utilization of Subtropical Agro-bioresources, College of Agriculture, Guangxi University, Nanning, People’s Republic of China; Vanderbilt University Medical Center

**Keywords:** two-component signal transduction system, *Xanthomonas*, global regulatory networks, pathogenicity regulation, transcription factors

## Abstract

*Xanthomonas* is a notorious plant pathogen causing serious diseases in hundreds of plant hosts. *Xanthomonas* species are equipped with an array of signal transduction systems that regulate gene expression to survive in various harsh environments and successfully infect hosts. Although certain pathogenicity-associated regulators have been functionally characterized, signal transduction systems always function as a regulatory network which remains to be elucidated in *Xanthomonas*. This study used a systematic approach to characterize all identified pathogenicity-associated regulators in Xanthomonas oryzae pv. oryzae (Xoo), including a transcriptional regulator with unknown function, and their interactive regulatory network. RNA sequencing was used in elucidating the patterns of the 10 pathogenicity-associated regulators identified. Results revealed that each pathogenicity-associated regulator has cross talk with others and all these regulators function as a regulatory network, with VemR and PXO_RS20790 being the master pathogenicity-associated regulators and HrpX being the final executant. Moreover, regulome analysis showed that numerous genes other than genes in pathogenicity islands are finely regulated within the regulatory network. Given that most of the pathogenicity-associated regulators are conserved in *Xanthomonadales*, our findings suggest a global network of gene regulation in this evolutionarily conserved pathogen. In conclusion, our study provides essential basic information about the regulatory network in Xoo, suggesting that this complicated regulatory network is one of the reasons for the robustness and fitness of *Xanthomonas* spp.

**IMPORTANCE** The host plant infection process of pathogenic bacteria is a coordinating cellular behavior, which requires dynamic regulation at several levels in response to variations in host plants or fluctuations in the external environment. As one of the most important genera of plant-pathogenic bacteria, *Xanthomonas* has been studied as a model. Although certain pathogenicity-associated regulators have been functionally characterized, interactions among them remain to be elucidated. This study systematically characterized pathogenicity-associated regulators in Xoo and revealed that cross talk exists among pathogenicity-associated regulators and function as a regulatory network in which a hierarchy exists among the regulators. Our study elucidated the landscape of the pathogenicity-associated regulatory network in *Xanthomonas*, promoting understanding of the infection process of pathogenic bacteria.

## INTRODUCTION

*Xanthomonas*, belonging to the *Gammaproteobacteria* subdivision of *Proteobacteria*, is a large genus of Gram-negative bacterial plant pathogens (1). *Xanthomonas* causes serious diseases in approximately 124 monocotyledonous and 268 dicotyledonous plants (2). *Xanthomonas* spp. occupy the fourth, fifth, and sixth positions in the list of the top 10 important scientific and economic bacterial plant pathogens put together by a consensus panel of bacterial pathologists (3). Bacterial leaf blight, caused by Xanthomonas oryzae pv. oryzae (Xoo), is found in tropical and temperate regions and has reduced yields by 10% to 50% in some areas (3, 4).

Bacteria harbor an array of signal transduction systems linking environmental stimuli to gene expression changes, ultimately allowing them to respond to their surroundings and survive and reproduce in various environments (5). Two-component signal transduction systems (TCSs) are predominant strategies by which bacteria sense and adapt to changing environments. A prototypical TCS comprises a histidine kinase (HK) sensor protein which can be autophosphorylated when recognizing a signal and a response regulator (RR) being phosphorylated and activated by a cognate HK (6). The activated RR then regulates gene transcription, enzyme activity, and/or protein-protein interactions, resulting in an appropriate response (7). Transcriptional factors comprise approximately 66% of all investigated bacterial RRs (8). Transcription factors, as “two-headed molecules,” play an important role in the linking of extracellular stimulus perception with intracellular stimuli in the switch governing the expression or repression of genes, operons, and regulons.

The members of the genus *Xanthomonas* are equipped with a large number of TCSs to cope with various hostile environments, and comparative genomic analyses showed that the total number of nucleotides from TCS genes constitute approximately 2.38% to 3.24% of the whole chromosome investigated (5, 9). *Xanthomonas* spp. also harbor a large number of transcription factors apart from TCSs.

Stimulated by a host plant environment or a special medium, RR HrpG is activated by its cognate HK (HpaS in Xanthomonas campestris pv. campestris) (10). The activated HrpG then regulates the expression of the key transcriptional regulator *hrpX* and other hypersensitive responses and pathogenicity (*hrp*) genes (11, 12). The absence of HrpG or HrpX totally abolishes the pathogenicity of *Xanthomonas* (13, 14). *Xanthomonas* spp. synthesize quorum-sensing molecules that increases with population size and regulates pathogenicity gene expression. TCS RpfC/RpfG couples the quorum sensing signaling system and intracellular regulatory networks through a second messenger cyclic di-GMP and the global transcriptional regulator Clp in X. campestris (15). RpfC/RpfG and Clp are essential for the full virulence of *Xanthomonas* (16–18). Furthermore, a large number of regulators are involved in multifaceted physiological metabolism in *Xanthomonas*. Ion homeostasis is detrimental to bacterial life, and many ion homeostasis regulatory systems regulate virulence in *Xanthomonas*. TCS ColS/ColR (also named VgrS/VgrR) directly senses extracytoplasmic and intracellular iron to control adaptation to stress due to iron depletion and virulence in *Xanthomonas* (19–21). PhoR/PhoB is a TCS regulating phosphate homeostasis in bacteria. In our previous study, we found that the absence of HK PhoR results in the constitutive expression of PhoB and reduces the virulence of Xoo (22). The transcriptional regulator Zur modulates the zinc homeostasis, exopolysaccharide (EPS) production, *hrp* gene expression, and virulence in X. campestris pv. campestris (23, 24). GntR family transcription regulator HpaR1 positively regulates xanthan polysaccharide production, extracellular enzyme activity, cell motility, tolerance to various stresses, hypersensitive reaction, and pathogenicity in X. campestris pv. campestris (25, 26). Moreover, orphan RR VemR regulates the exopolysaccharide production, motility, and virulence of X. campestris pv. campestris (27, 28). Although several regulatory systems have been studied, previous studies focused on them individually. However, bacterial signal transduction systems work as regulatory networks to coregulate groups, clusters, and their overlapping genes (29). The regulatory relationships and interactions among different regulators remain elusive in Xoo, and the landscape of the pathogenicity-associated regulatory network in *Xanthomonas* remains to be elucidated.

In this study, we investigated the pathogenicity function of all potential TCSs and transcription factors in Xoo PXO99^A^ and identified a pathogenicity-associated regulator that had not been characterized. Transcriptome sequencing (RNA-Seq) was then performed for the charting of the gene regulation patterns of the 10 pathogenicity-associated regulators identified. The landscape of the pathogenicity-associated regulatory network was elucidated.

## RESULTS

### Pathogenicity-associated regulators in Xanthomonas oryzae pv. oryzae.

**FIG 1:**
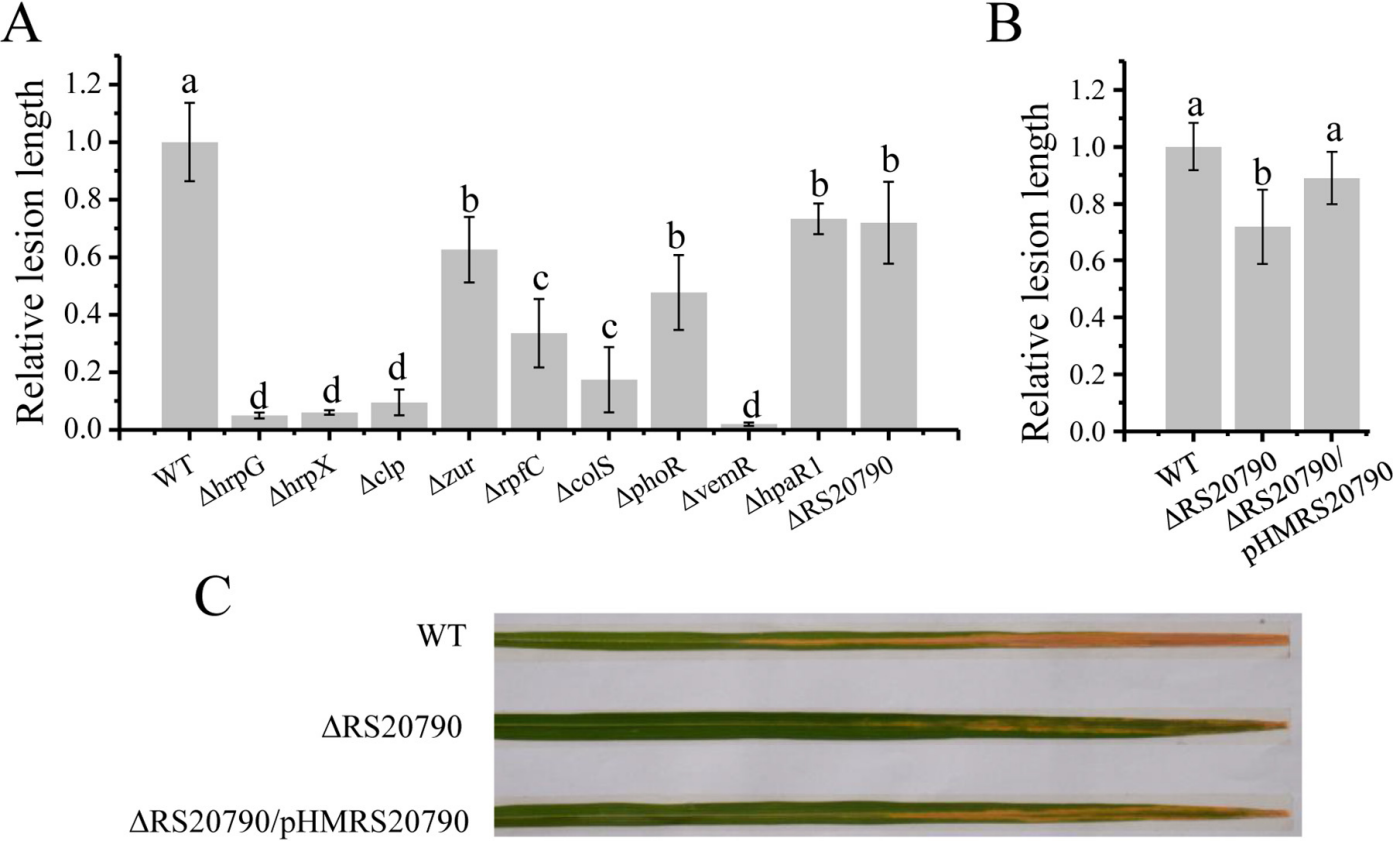
Relative pathogenicity of pathogenicity-associated regulator-deleted Xanthomonas oryzae pv. oryzae (Xoo) mutants. (A) Lengths of rice leaf lesions caused by the regulator-deleted mutants and wild-type (WT) Xoo were measured at 14 days after infection. The ratio between the lesion length caused by mutants and those by wild-type Xoo strains was defined as relative pathogenicity. Tukey test for analysis was used after ANOVA. Mutants caused a relative lesion length of ≤0.75 with a *P* value of ≤0.01 are shown in this figure. (B) Gene complementation assay of the Δ*RS20790* mutant. The relative lesion length of infected rice leaves was caused by Δ*RS20790*, complementation strains, and wild-type Xoo. (C) Blight symptoms of representative infected rice leaves.

To determine the pathogenicity-associated regulators in Xoo, we predicted 42 unique HK genes with the P2CS database and successfully obtained 39 HK gene-deleted mutants in Xoo PXO99^A^ (5). Here, we assayed the pathogenicity of the 39 mutants on a susceptible rice breed. Mutants presenting a relative lesion length of ≤0.75 were selected for further study (see Fig. S1A in the supplemental material). This analysis revealed that the deletion of *rpfC* (*PXO_RS08280*), *colS* (*PXO_RS18935*), and *phoR* (*PXO_RS19875*) significantly attenuated the pathogenicity of Xoo (Fig. 1). Apart from the HK genes, six orphan RRs were deleted individually, and their pathogenicity was investigated (Fig. S1B). The absence of HrpG (PXO_RS18055), the master regulator of *hrp* genes (11), and VemR (PXO_RS11975), which was reported as pathogenicity-associated RR in X. campestris (27), totally deprived Xoo of pathogenicity.

10.1128/mSystems.00789-20.1 FIG S1 Relative lesion lengths of infected rice leaves caused by histidine kinase mutants (A), orphan response regulator mutants (B), and transcriptional factor mutants (C). The wild-type Xoo and mutant strains were inoculated onto 10 leaves in five rice plants with at least three biological repeats. Lesion lengths caused by every strain were compared to the lesion lengths of wild-type PXO99^A^ and defined as relative lesion lengths. Mutants presenting a relative lesion length of ≤0.75 were selected for further study. Download FIG S1, TIF file, 0.6 MB. Copyright © 2021 Zheng et al. 2021 Zheng et al. https://creativecommons.org/licenses/by/4.0/ This content is distributed under the terms of the Creative Commons Attribution 4.0 International license.

A total of 97 genes were annotated as transcription factors in the genome of Xoo PXO99^A^ (GCA_000019585.2), 83 of which were successfully deleted. A pathogenicity assay was then performed (Fig. S1C). In addition to HrpX (PXO_RS18050), Clp (PXO_RS02870), Zur (PXO_RS06520), and HpaR1 (PXO_RS13460) which regulate the pathogenicity of *Xanthomonas* and transcription factor PXO_RS20790 of unknown function showed important roles in the pathogenicity of Xoo (Fig. 1A). Given that PXO_RS20790 was previously uncharacterized, the complementation of *PXO_RS20790* deleted mutant *ΔRS20790* was conducted. As shown in Fig. 1B and C, the expression of *PXO_RS20790* under the control of their native promoters in the cosmid pHM1 rescued the pathogenicity attenuation of *ΔRS20790* mutants. The pathogenicity attenuation of the *ΔphoR* mutant was previously rescued by the ectopic expression of *phoR* controlled by its native promoter (22). To further confirm the function of the other eight regulators in Xoo, we performed gene complementation on the other eight mutants. The pathogenicity of the mutants was rescued by these eight regulators, respectively (Fig. S2). Ten pathogenicity-associated regulators, including PXO_RS20790 of unknown function, were used in studying the pathogenicity-associated regulatory network in *Xanthomonas*.

10.1128/mSystems.00789-20.2 FIG S2 Gene complementation assay of Δ*vemR*, Δ*hrpX*, and Δ*hrpG* mutants (A), Δ*clp* and Δ*colS* mutants (B), Δ*hpaR1* mutant (C), Δ*zur* mutant (D), and Δ*rpfC* mutant (E). For *hrpX*, *hrpG*, and *hpaR1*, target genes and their native promoters and terminators were amplified using primers listed in Table S1 in the supplemental material and cloned to cosmid pHM1, generating pHMhrpX, pHMhrpG, and pHMhpaR1. For *vemR* and *zur*, promoter and open reading frame region were amplified, separated, and cloned to pHM1 via three restriction sites, resulting pHMvemR and pHMzur. For *clp* and *colS*, target genes and their native promoters and terminators were cloned pHM1 via *in vitro* homologous recombination using One Step cloning kit (Vazymebiotech, Nanjing, China). The rescue recombinant plasmids were then transferred to the corresponding mutants for gene complementation assay. For Δ*rpfC* mutant, the deleted DNA with its flanking DNA were amplified from the wild-type strain and cloned to the suicide plasmid pK18mobsacB, and the deleted DNA was knocked back into the Δ*rpfC* mutant for gene complementation assay. Download FIG S2, TIF file, 1.7 MB. Copyright © 2021 Zheng et al. 2021 Zheng et al. https://creativecommons.org/licenses/by/4.0/ This content is distributed under the terms of the Creative Commons Attribution 4.0 International license.

### Conservation of pathogenicity-associated regulators within *Xanthomonadales*.

**FIG 2:**
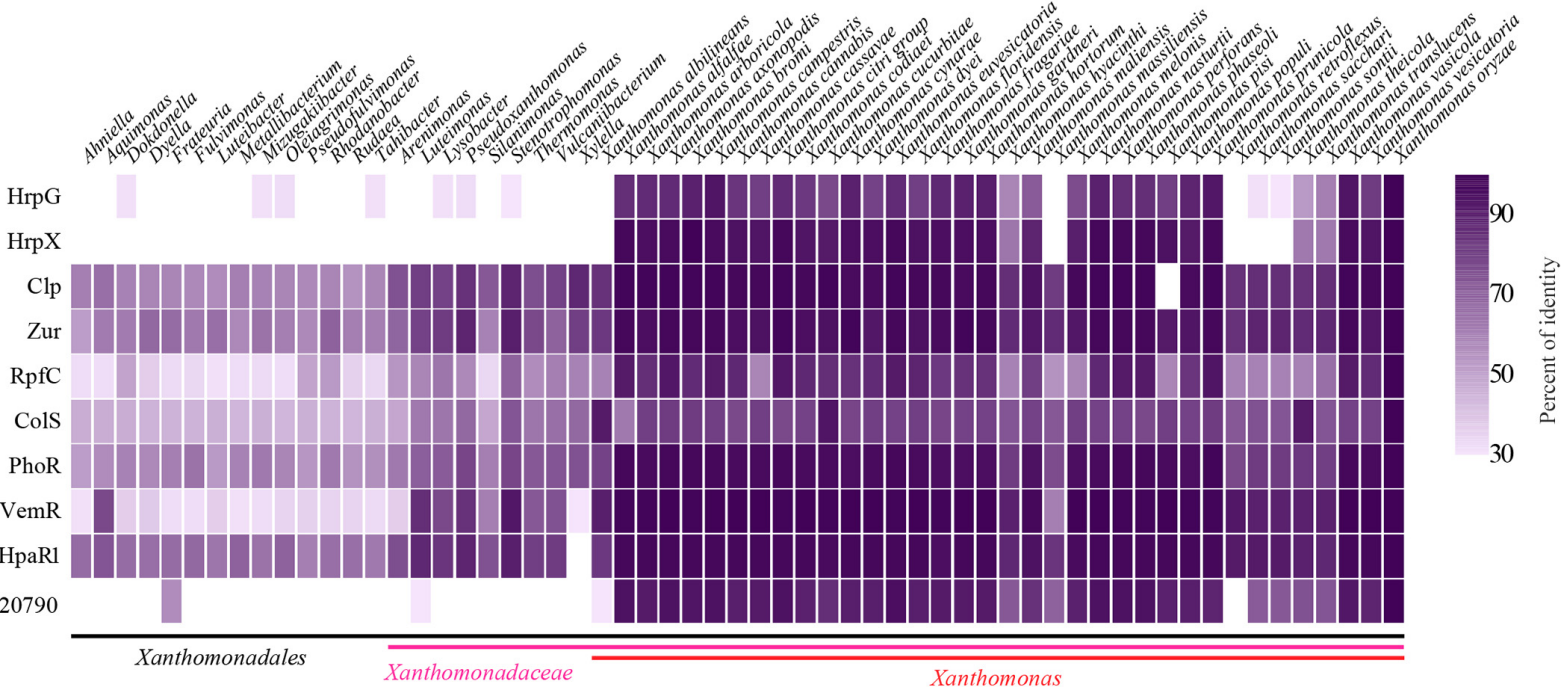
Conservation analysis of pathogenicity-associated regulators of Xanthomonas oryzae pv. oryzae (Xoo). The percent identity of pathogenicity-associated regulators orthologs of the best hit in selected taxonomy, compared with pathogenicity-associated regulators of Xoo, is indicated in each purple square. Pathogenicity-associated regulators are indicated on the left. Organisms are ordered on the basis of taxonomy.

To investigate the conservation of pathogenicity-associated regulators, we identified the homologs of HrpG, HrpX, Clp, Zur, RpfC, ColS, PhoR, VemR, HpaR1, and PXO_RS20790 in every genus within *Xanthomonadales* and every species within *Xanthomonas* by using a BLASTp search against the NCBI protein reference sequence database. The amino acid percent identity of the best hit in the selected taxonomy is shown in Fig. 2. HrpG and HrpX, which control all the *hrp* genes, were highly conserved in most species within *Xanthomonas* genus, except in Xanthomonas albilineans and Xanthomonas sacchari, which are unique phytopathogenic *Xanthomonas* species lacking *hrp* gene cluster (30–32), and nonpathogenic species, including Xanthomonas massiliensis, Xanthomonas retroflexus, and Xanthomonas sontii (33–35). PXO_RS20790 was conserved in *Xanthomonas* and found in *Frateuria* and *Arenimonas*. Other pathogenicity-associated regulators were conserved in almost all *Xanthomonadales* bacteria, including nonpathogenic bacteria, but HpaR1 was missing in *Xylella* genus and Clp was not found in Xanthomonas pisi, suggesting their important function beyond pathogenicity.

### Cross talk among pathogenicity-associated regulators.

**FIG 3:**
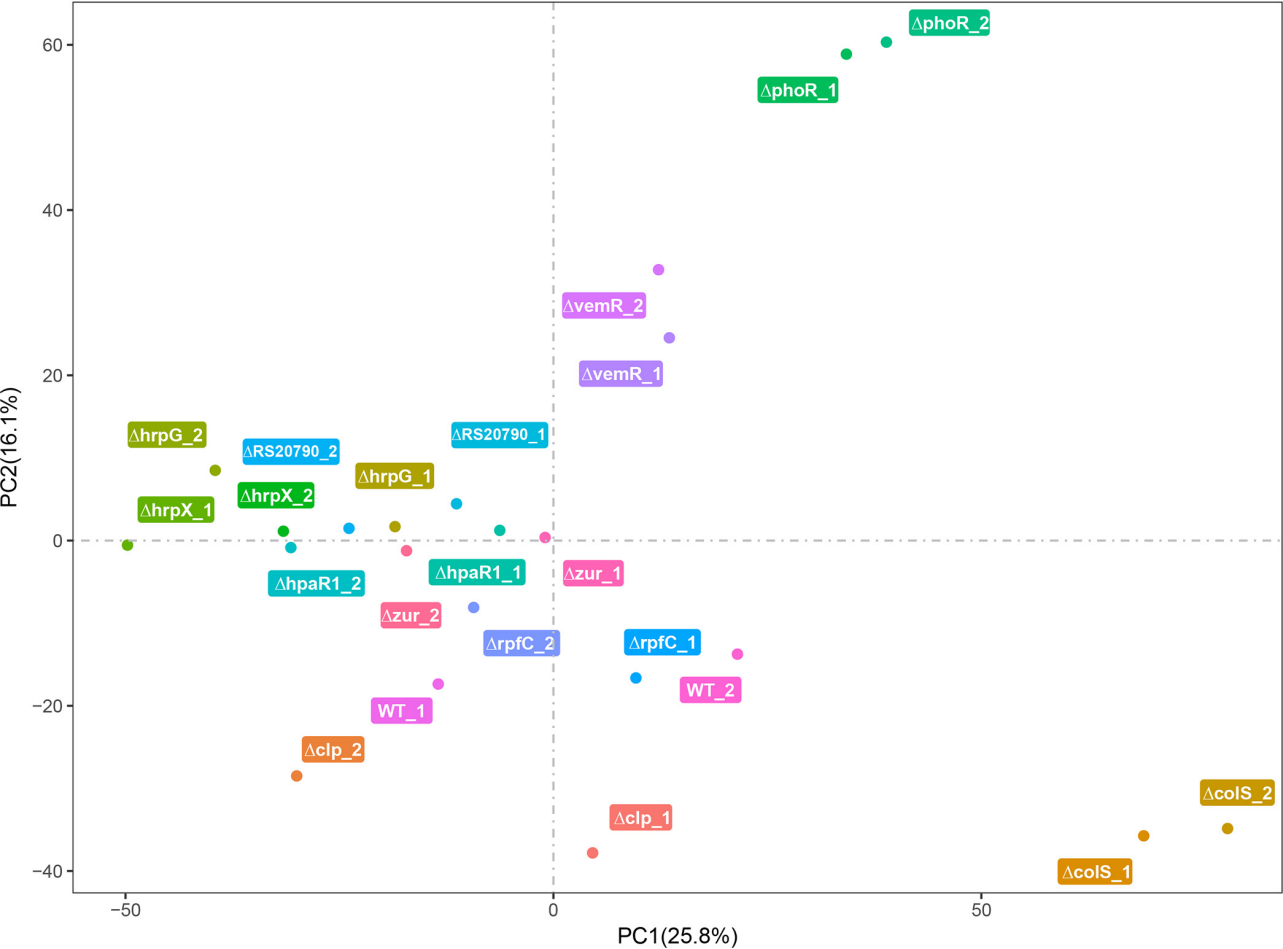
Principal-component analysis (PCA) based on the fragments per kilobase of exon model per million reads mapped (FKPM) of mutants and wild-type Xanthomonas oryzae pv. oryzae strains.

To chart the gene expression landscape of pathogenicity-associated regulator mutants, we conducted RNA-seq analysis. All the 10 Xoo mutants with weakened pathogenicity and wild-type Xoo PXO99^A^ were cultured in the pathogenicity gene-induced medium XOM2 to the middle logarithmic phase, followed by RNA extraction, cDNA preparation, and sequencing. Principal-component analysis (PCA) was performed using the fragments per kilobase of exon model per million reads mapped (FPKM) of mutants and wild-type Xoo strains for the visual assessment of gene expression similarities and differences among the Xoo strains (Fig. 3). As shown in Fig. 3, *phoR* (*ΔphoR*) and *colS* (*ΔcolS*) mutants showed discrete clustering from other mutants, indicating that the gene expression of *ΔphoR* and *ΔcolS* mutants is overall significantly different from that in other Xoo strains. Consistent with the result of PCA analysis, *phoR* deletion weakened the growth of Xoo most sharply (Fig. S3A). ColS/ColR regulates the tolerance of environmental stress factors, including phenol, copper, and hydrogen peroxide (20). Copper tolerance assay showed that *colS* deletion attenuated copper tolerance most prominently, except for the growth-related regulators PhoR and VemR (Fig. S3B).

10.1128/mSystems.00789-20.3 FIG S3 Growth in XOM2 medium (A) and copper tolerance (B) of pathogenicity-associated regulator mutants. (A) The NB cultured Xoo strains were inoculated onto XOM2. The absorbance at λ = 600 was measured at certain times, and the values are expressed as means ± standard deviations. Every Xoo strain was performed for three biological repeats. (B) The wild-type Xoo and mutant strains were inoculated to NB agar medium with or without 0.3mM Cu^2+^. The copper tolerance of each Xoo strain was estimated by the growth on NB agar medium with 0.3 mM Cu^2+^ 48 h postinoculation. Download FIG S3, TIF file, 1.4 MB. Copyright © 2021 Zheng et al. 2021 Zheng et al. https://creativecommons.org/licenses/by/4.0/ This content is distributed under the terms of the Creative Commons Attribution 4.0 International license.

**FIG 4:**
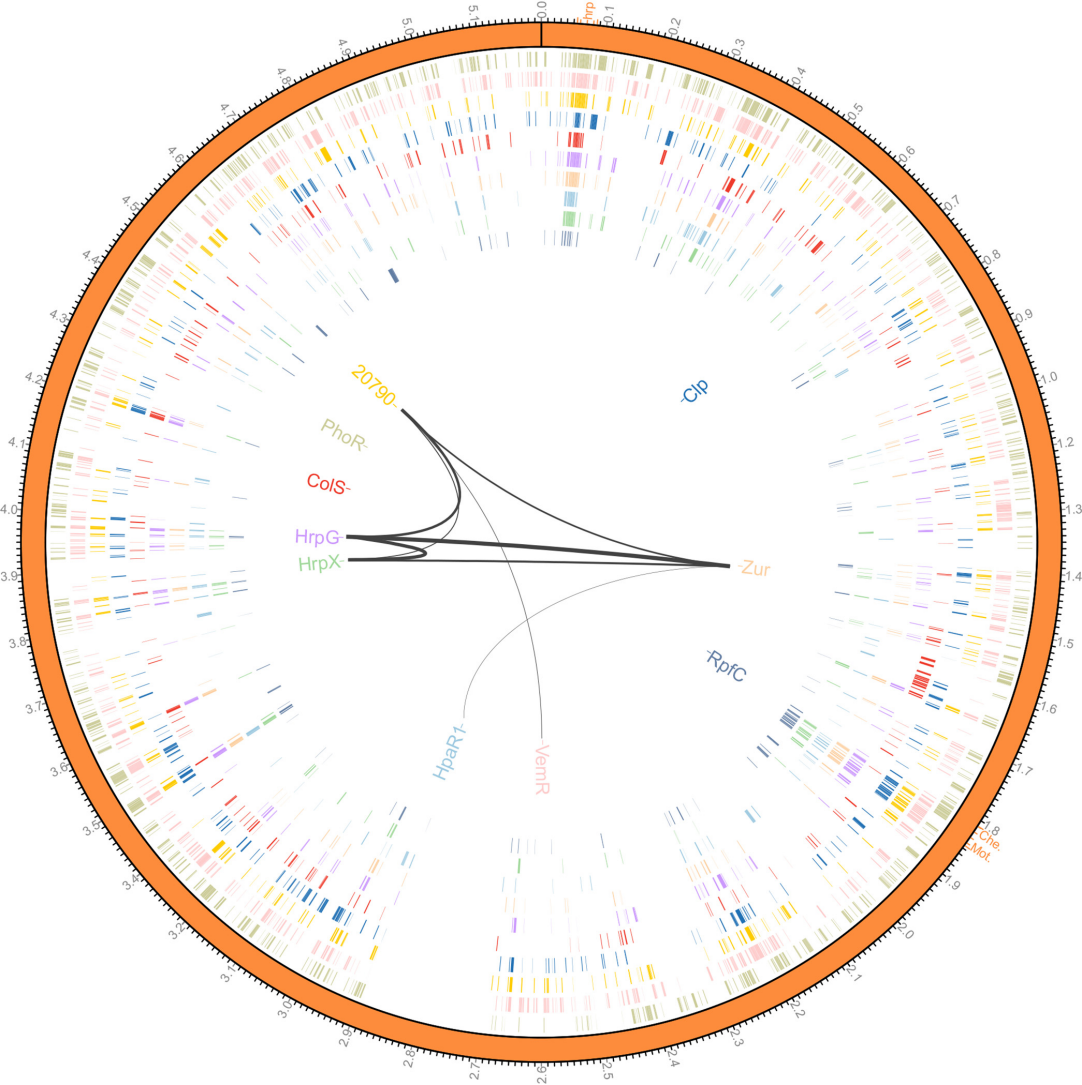
Pathogenicity-associated regulome of Xanthomonas oryzae pv. oryzae (Xoo). The first circle represents the genome of Xoo PXO99^A^. The *hrp* gene cluster and chemotaxis and mobility gene cluster are highlighted in this circle. The inner 10 circles highlight the target genes regulated by pathogenicity-associated regulators (PhoR, VemR, PXO_RS20790, Clp, ColS, HrpG, Zur, HpaR1, HrpX, and RpfC from outside to inside). The innermost diagram depicts the top eight cross talk between the pathogenicity-associated regulators. The thickness of curves in the innermost diagram indicates the ratio of shared target genes to the total target genes of every two pathogenicity-associated regulators.

The differentially expressed genes (DEGs) caused by the absence of pathogenicity-associated regulators were then analyzed using DESeq (using a false discovery rate [FDR]-adjusted *P* value of <0.05). The number of DEGs dramatically varied among pathogenicity-associated regulator mutants. PhoR and VemR regulated a wide range of genes (911 and 862, respectively), whereas the target genes of RpfC, HrpX, and HpaR1 were less than 200 (Table 1). Then, we quantitatively analyzed the regulons’ cross talk. As shown in Table 1, each pathogenicity-associated regulator shared regulon members with others, indicating that each pathogenicity-associated regulatory circuit connects with others. To quantify the cross talk among the pathogenicity-associated regulators, we ranked the cross talk by the ratio of shared target genes to the total target genes of every two pathogenicity-associated regulators. The top eight cross talk between pathogenicity-associated regulators was depicted using curves with different thickness in Fig. 4. Approximately 97.6% (163/167) of the target genes of HrpX were regulated by HrpG. Furthermore, most of the HrpG- and HrpX-regulated genes were controlled by VemR (241/278 and 157/163, respectively) and PXO_RS20790 (252/278 and 156/163, respectively). Apart from *hrp* genes, VemR and PXO_RS20790 regulate a considerable number of target genes of other pathogenicity-associated regulators (Table 1). This finding indicates that VemR and PXO_RS20790 are master pathogenicity-associated regulators with a large degree of cross talk with other pathogenicity-associated regulators.

**TABLE 1:** Quantitative analysis of the target genes of each pathogenicity-associated regulator

Pathogenicity-associated regulator	No. of target genes^*a*^									
	HrpG	HrpX	Clp	Zur	RpfC	ColS	PhoR	VemR	HpaR1	PXO_RS20790
HrpG	**278**	163	117	187	86	79	140	241	95	252
HrpX	163	**167**	85	127	64	59	90	157	72	156
Clp	117	85	**387**	93	79	70	133	190	63	138
Zur	187	127	93	**227**	84	68	132	203	92	203
RpfC	86	64	79	84	**135**	31	47	111	50	102
ColS	79	59	70	68	31	**275**	105	141	53	86
PhoR	140	90	133	132	47	105	**911**	340	89	198
VemR	241	157	190	203	111	141	340	**862**	122	320
HpaR1	95	72	63	92	50	53	89	122	**178**	108
PXO_RS20790	252	156	138	203	102	86	198	320	108	**428**

a The intersecting values show the number of target genes shared by two pathogenicity-associated regulators.

### Pathogenicity-associated regulators function as a regulatory network.

**FIG 5:**
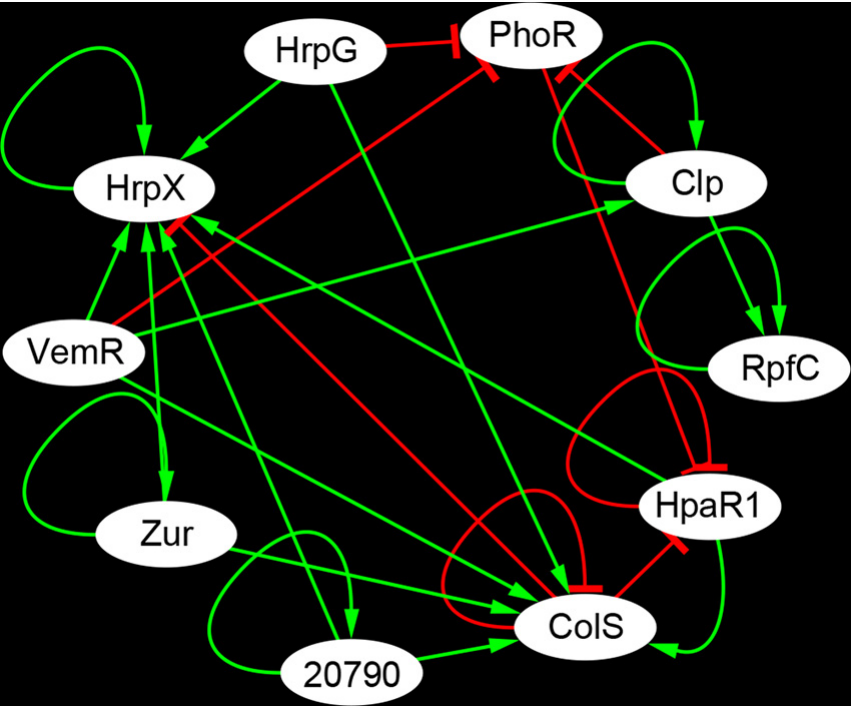
Regulatory relationships among pathogenicity-associated regulators from gene expression data. Green lines with arrowheads represent positive regulation. Red “T” lines indicate negative regulation.

The regulatory relationships between every two pathogenicity-associated regulators were analyzed and visualized using Cytoscape. As shown in Fig. 5, HrpX, Zur, PXO_RS20790, RpfC, and Clp were positively autoregulated, whereas ColS and HpaR1 were negatively autoregulated. PhoR was under the negative control of several regulators, including HrpG, VemR, and Clp. This finding is consistent with our previous one showing that PhoBR expression should be accurately controlled for normal physiological function (22). HrpG, VemR, Zur, and PXO_RS20790 regulated the expression of at least two pathogenicity-associated regulators but were not subject to the regulation of others. HrpX was subject to the positive control of HrpG, VemR, Zur, PXO_RS20790, HpaR1, and HrpX itself and negatively controlled by ColS, implying that HrpX is the final executant of pathogenicity regulation. To verify the regulatory relationship, we assayed the interaction between transcription factors and the promoter DNAs of their target genes with a bacterial one-hybrid system (36). As shown in Fig. S4, HrpG interacted with the promoter DNAs of *hrpX*, *phoR*, and *colS*, indicating that these genes may be directly regulated by HrpG. The interactions between the regulators indicated that pathogenicity-associated regulators function as a regulatory network, and a hierarchy exists among the regulators. HrpG, VemR, Zur, and PXO_RS20790 may be at architecturally high levels in the hierarchy of this pathogenicity-associated network, whereas HrpX may be at a low level. The hierarchy of the pathogenicity-associated network provides a cell with adequate flexibility to tune its transitions.

10.1128/mSystems.00789-20.4 FIG S4 Interaction between HrpG and the promoter DNAs of *hrpX*, *phoR*, and *colS*. The interaction between HrpG and its target DNAs was determined by bacterial one-hybrid reporter system. The open reading frame of *hrpG* was cloned into pTRG via *Bam*HI and *Xho*I. The promoter DNAs of *hrpX*, *phoR*, and *colS* were cloned into pBXCMT via *in vitro* homologous recombination using One Step cloning kit (Vazymebiotech, Nanjing, China) using primers listed in Table S1. A pair of pBXCMT/pTRG plasmids were cotransformed into the reporter strain XL1-Blue. Growth of reporter strains was tested together with the self-activation controls on a selective medium containing 3-amino-1,2,4-triazole (3-AT) and streptomycin to determine the interaction. Download FIG S4, TIF file, 1.6 MB. Copyright © 2021 Zheng et al. 2021 Zheng et al. https://creativecommons.org/licenses/by/4.0/ This content is distributed under the terms of the Creative Commons Attribution 4.0 International license.

### Pathogenicity-associated regulome.

Overall, 1,766 genes (40.4% of all predicted genes in the genome of Xoo PXO99^A^) were differentially expressed in the absence of at least one pathogenicity-associated regulator; 796 of the genes were subject to the regulation of more than one regulator. As depicted in Fig. 4, target genes were more densely distributed in the *hrp* gene cluster (56.1 to 86.3 kb, *PXO_RS00290* to *PXO_RS00425*) and the gene cluster encoding chemotaxis- and mobility-associated proteins (1.81 to 1.85 Mb, *PXO_RS08335* to *PXO_RS08470*). The *hrp* gene cluster encoding the type III protein secretion system is the most important pathogenicity island required for pathogenicity in *Xanthomonas* spp. and other bacterial pathogens (37, 38). It was tightly controlled by nearly all of the pathogenicity-associated regulators investigated in this study. Three *hrp* genes, *PXO_RS00320*, *PXO_RS00325*, and *PXO_RS00360*, were coregulated by all 10 regulators. Sixteen *hrp* genes were regulated by 9 of the 10 pathogenicity-associated regulators (see Table S2 in the supplemental material). This result indicates that *hrp* genes are the regulatory focus of pathogenicity-associated regulators. The literature showed that expression of Xoo chemotaxis and motility components is induced by plant conditions and is required for entry, colonization, and pathogenicity (39). Target gene distribution highlights the importance of chemotaxis and mobility, which are fine-tuned by many pathogenicity-associated regulators (Fig. 4). Moreover, hypothetical protein (PXO_RS17445) and cellulase (PXO_RS23345) were also regulated by the 10 regulators. The type II system secreted cell wall-degrading enzymes including cellulase play an important role in the interaction between Xoo and its host plant (40, 41). In addition to the *hrp* gene cluster and chemotaxis and mobility gene cluster, pathogenicity-associated regulome genes were distributed over the whole genome of Xoo (the empty region of 2.7 to 2.9 Mb is the result of near-perfect tandem duplication of 212 kb [42]). This result suggests that numerous genes other than the regulated pathogenicity island genes contribute to the pathogenicity of *Xanthomonas*.

10.1128/mSystems.00789-20.7 TABLE S2 Target genes regulated by pathogenicity-associated regulators. Download Table S2, XLSX file, 0.25 MB. Copyright © 2021 Zheng et al. 2021 Zheng et al. https://creativecommons.org/licenses/by/4.0/ This content is distributed under the terms of the Creative Commons Attribution 4.0 International license.

### Functional profiling of pathogenicity-associated regulatory network.

**FIG 6:**
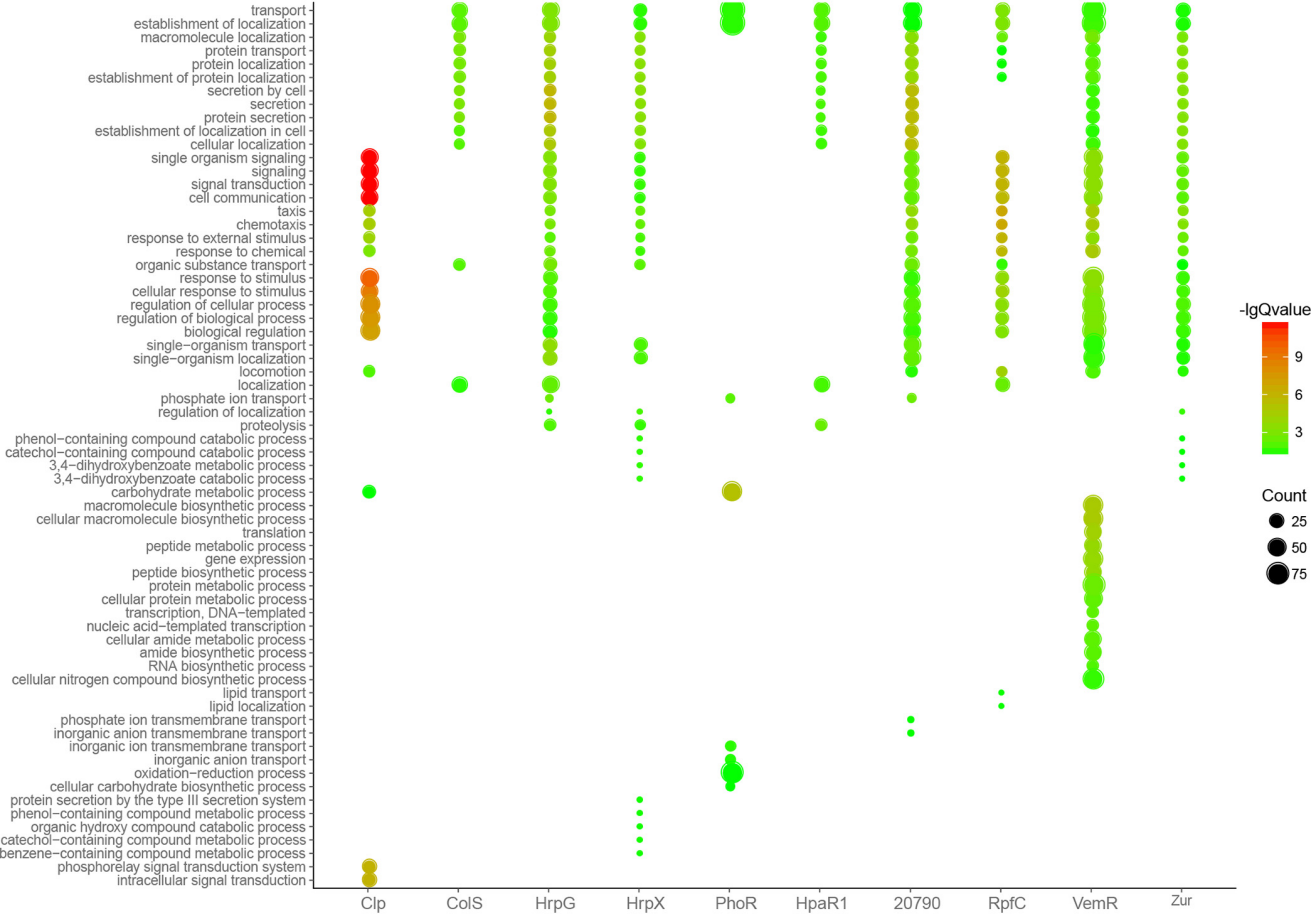
Functional profiling of pathogenicity-associated regulatory network of Xanthomonas oryzae pv. oryzae. Gene ontology (GO) enrichment analysis for individual regulons was conducted, and significantly overrepresented biological process GO terms with Q values of <0.05 were displayed.

To profile the function of the pathogenicity-associated regulome, we conducted gene ontology (GO) enrichment analysis for individual regulons. The significantly overrepresented biological process GO terms are displayed in Fig. 6. The target genes of HrpG, HrpX, PXO_RS20790, RpfC, VemR, and Zur shared many enriched GO items. GO terms associated with protein secretion, localization and transport, chemotaxis, signaling, and response to stimulus were significantly enriched in the regulons of HrpG, PXO_RS20790, and Zur. In contrast, GO terms associated with response to stimulus were not enriched in the regulon of HrpX, whereas GO terms associated with protein secretion were not present in the RpfC regulon. Besides the GO terms enriched in HrpG, PXO_RS20790, and Zur, GO terms associated with gene translation and transcription were significantly overrepresented in the regulon of VemR, implying its important role in the regulation of fundamental cellular activities. The regulons of ColS and HpaR1 shared most of their enriched GO terms, including protein secretion, localization, and transport. The GO enrichment analysis results of Clp and PhoR regulons were distinct from those of the others. Clp mainly regulated the expression of genes associated with signal transduction, chemotaxis, and response to stimulus. Consistent with our previous finding (22), PhoR regulon consisted of nutrient transport- and metabolism-associated genes.

## DISCUSSION

As one of the most important genera of plant-pathogenic bacteria, *Xanthomonas* has been studied as a model. Although its signal transduction systems have been functionally characterized, interactions among them remain to be elucidated. This study investigated the pathogenicity functions of the TCSs and transcription factors in Xoo PXO99^A^. A total of 128 regulators (39 HKs, 6 orphan RRs, and 83 transcription factors) were screened, but only 10 were found to be essential for the full pathogenicity of Xoo. The absence of individual regulator genes did not alter the pathogenicity of Xoo possibly because of genetic redundancy, that is, different genes have a common function, and the disruption of individual genes does not result in a discernible phenotype (43). Variations in host plants or fluctuations in the external environment may trigger different modes of redundant signal transduction in pathogens. The genetic redundancy of signaling systems could be one of the reasons for the robustness and fitness of *Xanthomonas* spp.

HrpG and HrpX are known as key regulators for the expression of *hrp* genes, which are involved in the construction of the type III secretion apparatus (44). HrpG/HrpX network has been reported in many species of *Xanthomonas*. Microarray analyses have shown that 123 genes overlapped in the regulons of HrpG and HrpX in Xanthomonas axonopodis pv. citri (11). We found 74 orthologous genes of these 123 genes in Xoo PXO99^A^ using BLASTn. More than half (45/74) of these genes are in the regulons of HrpG or HrpX revealed in this study (see Fig. S5 in the supplemental material). Chromatin immunoprecipitation-sequencing (ChIP-seq) identified 186 candidate HrpG downstream genes in X. campestris (45). However, just a few of these genes were overlapped with HrpG or HrpX regulons identified in this study. One probable reason may be that most of the HrpG regulon genes identified in this study were indirectly regulated by HrpG via other regulators like HrpX. HrpX was reported as the most downstream component of the *hrp* regulatory cascade in X. campestris pv. vesicatoria (46). This is consistent with the finding revealed in this study that HrpX was subject to the control of HrpG, VemR, Zur, ColS, PXO_RS20790, HpaR1, and HrpX itself.

10.1128/mSystems.00789-20.5 FIG S5 Venn diagram showing the overlapped genes in the HrpG/HrpX regulons revealed in this study and the Hrp regulon previously reported. Overlapped genes in HrpG and HrpX regulons identified in Xanthomonas axonopodis pv. citri 306 (11) were used as queries to identify orthologous genes in Xanthomonas oryzae pv. oryzae PXO99^A^ using BLASTn. Seventy-four genes in Xoo PXO99^A^ were identified as orthologs. These orthologs were then overlapped with the genes in HrpG and HrpX regulons identified in this study and visualized in Venn diagram. Download FIG S5, TIF file, 0.2 MB. Copyright © 2021 Zheng et al. 2021 Zheng et al. https://creativecommons.org/licenses/by/4.0/ This content is distributed under the terms of the Creative Commons Attribution 4.0 International license.

Pathogenicity-associated regulome analysis showed that *hrp* genes are under the control of almost all the pathogenicity-associated regulators and are the core target genes of the pathogenicity-associated regulome. Chemotaxis and the mobility gene cluster are the regulatory focus of most pathogenicity-associated regulators. Accessory target genes are scattered in the whole genome of Xoo. In addition to genomic variation, transcriptomic variability has a profound effect on the adaptation and fitness of bacteria (47). Bacterial regulatory networks can tolerate the addition of new target genes (48). This finding has made us think that *hrp*, chemotaxis, and mobility genes are the results of the long-term evolution of pathogenicity-associated regulatory networks, and the other scattered target genes are the results of coevolution between Xoo and fluctuant environments in host plants. Transcription activator-like effectors (TALEs), which act as transcriptional activators and reprogram plants for the benefit of the pathogen, are encoded by scattered genes. The TALE gene *PXO_RS08565* is positively regulated by most of the pathogenicity-associated regulators, including HrpG, HrpX, Zur, PhoR, VemR, HpaR1, and PXO_RS20790 (see Table S2 in the supplemental material). However, PXO_RS08545, a TALE pseudogene, is only positively controlled by HrpG and HrpX (Table S2). TALEs are numerous within Xoo and *X. oryzae* pv. oryzicola but few or even absent in other *Xanthomonas* species (49). This contrasting size of TALE repertoires is postulated as the result of different evolutionary forces acting on different *Xanthomonas* lineages (49, 50).

Clp is an effector of the conserved second messenger c-di-GMP (51). Zur, ColS, and PhoR are the key regulators tuning the homeostasis of zinc, iron, and phosphate, respectively (19, 22, 24). RpfC is an HK sensing and responding to quorum-sensing signals (52). VemR regulates flagellum-derived cell motility by interacting with sigma factor RpoN2 (28). Our analysis showed that Clp, Zur, RpfC, ColS, PhoR, VemR, and HpaR1 are conserved in almost all the investigated representatives of *Xanthomonadales*. This evolutionary conservation shows that biological processes modulated by these regulators are important to the survival of *Xanthomonadales* bacteria and undergo independent selective pressures. Clp which is downstream of RpfC regulates 299 genes including *zur* in X. campestris pv. campestris (53). However, this regulatory circuit was not revealed in the regulatory network built in this study. This might be the result of the regulatory pattern difference under the different growth conditions. More growth conditions should be employed to construct a more complete regulatory network in further study.

In summary, our findings revealed that pathogenicity-associated regulators function as a hierarchical regulatory network. HrpX, the final executant, may be at an architecturally low level in the hierarchy of this pathogenicity-associated network, whereas VemR and PXO_RS20790 may play a role as master pathogenicity-associated regulators and engage in cross talk with others. Moreover, this study reveals that important pathogenicity factors, including *hrp* genes, chemotaxis, and mobility, and cell wall-degrading enzymes, were fine-tuned by many pathogenicity-associated regulators.

## MATERIALS AND METHODS

### Primers, strains, and culture conditions.

Primers used in this study are listed in Table S1 in the supplemental material. Δ*rpfC*, Δ*colS*, Δ*phoR*, and other HK gene-deleted mutants of Xanthomonas oryzae pv. oryzae (Xoo) PXO99^A^ were obtained in our previous study (5). Other signal transduction systems were individually deleted. Xoo strains were grown in the NB medium (beef extract, 3 g/liter; yeast extract, 1 g/liter; polypeptone, 5 g/liter; and sucrose, 10 g/liter) or on the NB agar medium (NA) at 28°C, except in special circumstances as described in this section. Escherichia coli strains were cultured in the Luria-Bertani (LB) medium (54) or on the LB agar medium at 37°C. The final concentrations of antibiotics used were as follows: kanamycin, 25 μg/ml; spectinomycin, 50 μg/ml.

10.1128/mSystems.00789-20.6 TABLE S1 Primers used in this study. Download Table S1, XLSX file, 0.03 MB. Copyright © 2021 Zheng et al. 2021 Zheng et al. https://creativecommons.org/licenses/by/4.0/ This content is distributed under the terms of the Creative Commons Attribution 4.0 International license.

### Gene deletion.

Candidate HK and RR genes were predicted using P2CS (55), and candidate transcription factor genes were selected through the genome annotation of Xoo PXO99^A^ (GCA_000019585.2). Target genes in Xoo were deleted through allelic homologous recombination with a previously described method (5). In brief, the two flanking regions of the target gene were cloned to the suicide plasmid pK18mobsacB with three appropriate restriction sites and primers listed in Table S1. Primers 1F and 1R were used for homologous DNA fragment 1 PCR amplification, and primers 2F and 2R were used for homologous DNA fragment 2. PCR amplification was performed using TransFast *Taq* DNA polymerase (TransGen Biotech, Beijing, China) following the manufacturer’s instructions. The annealing temperature of PCR amplification was provided in Table S1. The recombinant plasmid was imported into the wild-type PXO99^A^ strain, and one crossover mutant was generated. The second crossover mutant was generated by culturing on the NAS medium (NA containing 10% sucrose). PCR screening was performed on the final gene deletion mutants.

### Gene complementation.

For complementation analysis, the open reading frame (ORF) region of the target gene and its promoter and terminator region were amplified and cloned to a broad-host-range cosmid pHM1. The recombinant vector was verified through Sanger sequencing and electroporated into the Xoo mutants for the analysis of complementation.

### Pathogenicity assay.

The pathogenicity of the Xoo strains was assayed through leaf clip inoculation (56). Susceptible cultivar rice MH63 (4 to 5 weeks) were used as the host plants (5). The Xoo strains were cultured to the late logarithmic growth phase. Xoo culture was then adjusted to an optical density at 600 nm (OD_600_) of 1.0 and inoculated into the rice leaves. The lesion lengths of the infected rice leaves were measured 14 days after inoculation. All the pathogenicity assays were repeated at least three times. In each experiment, 10 leaves in five plants were inoculated for each strain. Lesion length caused by every strain was compared to that of the wild-type PXO99^A^ and defined as the relative lesion length. Mutants presenting a relative lesion length of ≤0.75 were selected for further study. Tukey test for analysis after analysis of variance (ANOVA) was used for significant analysis. A *P* value of 0.01 was used in determining significance.

### Conservation analysis of regulators.

To identify orthologs, we used amino acid sequence of pathogenicity-associated regulators as queries of the nonredundant protein sequence database within every genus in *Xanthomonadales* and every *Xanthomonas* sp. The best BLASTp hits with ≥50% sequence coverage and ≥30% identity were identified as orthologous proteins in the selected taxonomy of *Xanthomonadales* (57). The percentage of each ortholog’s amino acid sequence identity, compared with regulators of Xoo PXO99^A^, was visualized as a heatmap.

### RNA sequencing.

Xoo strains were cultured in the *hrp* gene-induced medium XOM2 (d-xylose, 0.18%; d,l-methionine, 670 μM; sodium l-(+)-glutamate, 10 mM; KH_2_PO_4_, 14.7 mM; MnSO_4_, 40 μM; Fe(III)-EDTA, 240 μM; and 5 mM MgCl_2_) to the middle logarithmic growth phase, in which the OD_600_ values are 0.4 for the Δ*phoR* mutant and 0.8 for wild-type PXO99^A^ and Xoo mutants (22). Total RNA was extracted with an EasyPure RNA kit (TransGen Biotech, Beijing, China) according to the manufacturer’s instructions for Gram-negative bacteria and then digested with DNase I (Invitrogen, CA, USA) for the prevention of DNA contamination in the RNA samples. The RNA samples, two biological replicates per Xoo strains, were then subjected to rRNA removal, fragmentation, cDNA synthesis, and sequencing with Illumina Hiseq4000 at Novogene (Beijing, China).

### Data analysis and visualization.

Clean read data were obtained after RNA sequencing and data filtering. The expected number of FPKM was counted by Htseq (58). After matrixing, log_2_ (FPKM + 1) was then used for principal-component analysis (PCA) with the fast.prcomp function of R language. Gene expression in each Xoo mutant was compared with that in the wild-type strain with the R package Deseq (59). A gene with an adjusted *P* value of <0.05 and tested using a negative binomial distribution was defined as DEG (Table S2). The regulatory relationship among pathogenicity-associated regulators from gene expression data were visualized using Cytoscape (60), and the figure of pathogenicity-associated regulome was generated using Circos (61). The GO enrichment of DEG was analyzed and visualized using OmicShare, a free online platform for data analysis (http://www.omicshare.com/tools).
