# Supplementary figures and images for: Elucidation of the Pathogenicity-Associated Regulatory Network in Xanthomonas oryzae pv. oryzae
Source: mSystems. 2021 Mar 9;6(2):e00789-20. doi: 10.1128/mSystems.00789-20 (PMC8546981; doi:10.1128/mSystems.00789-20)

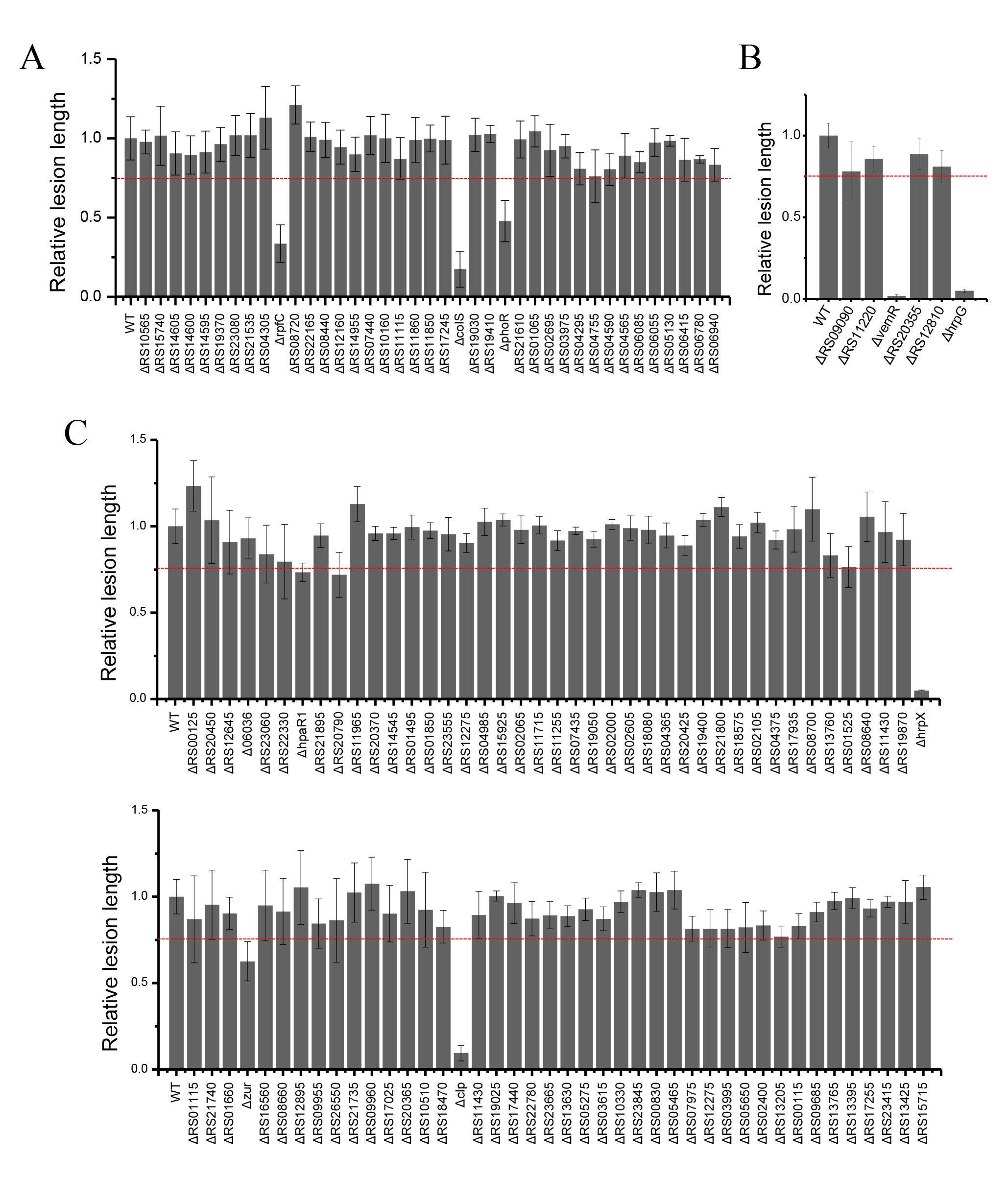

Supplement: FIG S1 [file msystems.00789-20-sf001.tif]

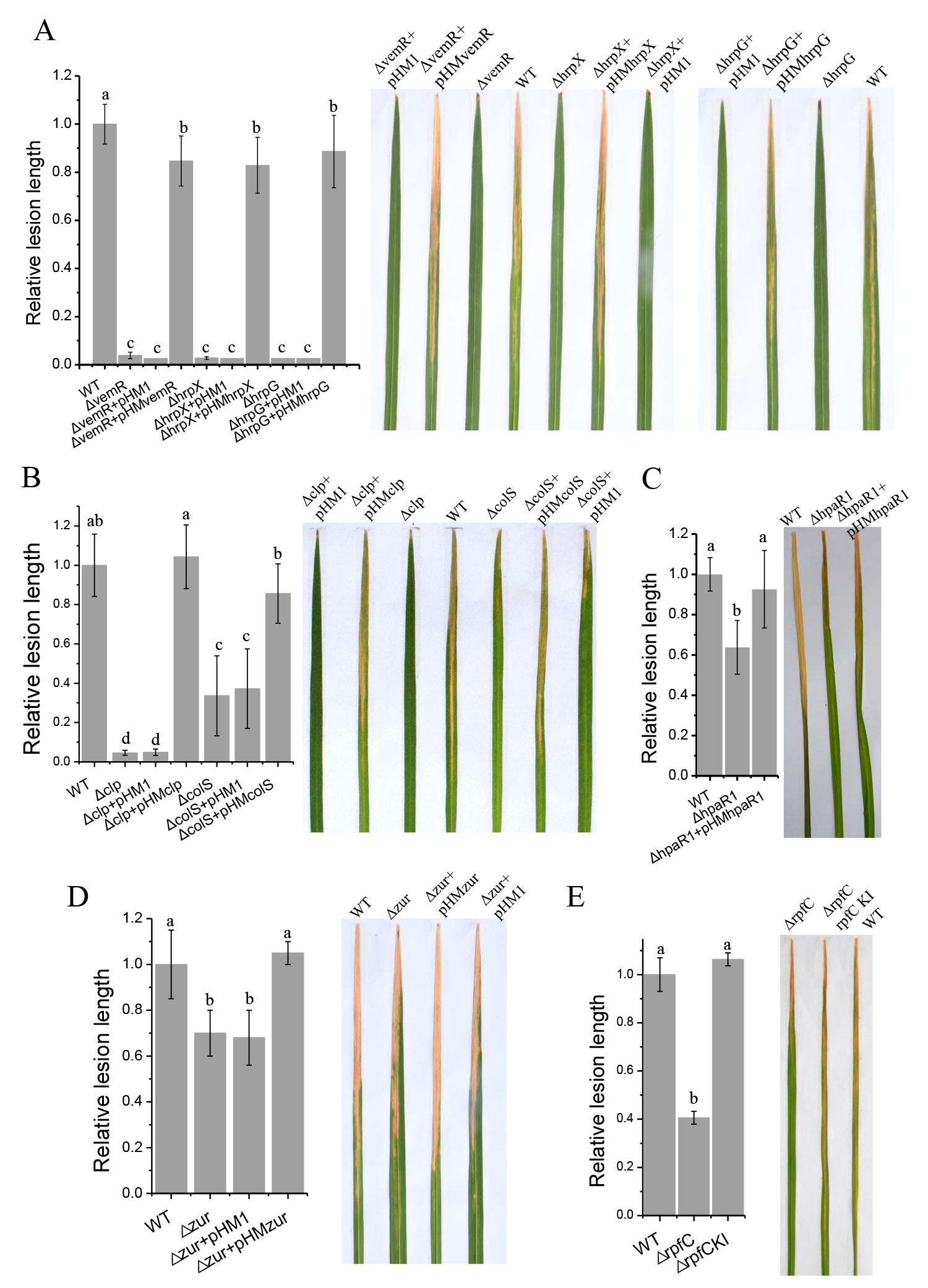

Supplement: FIG S2 [file msystems.00789-20-sf002.tif]

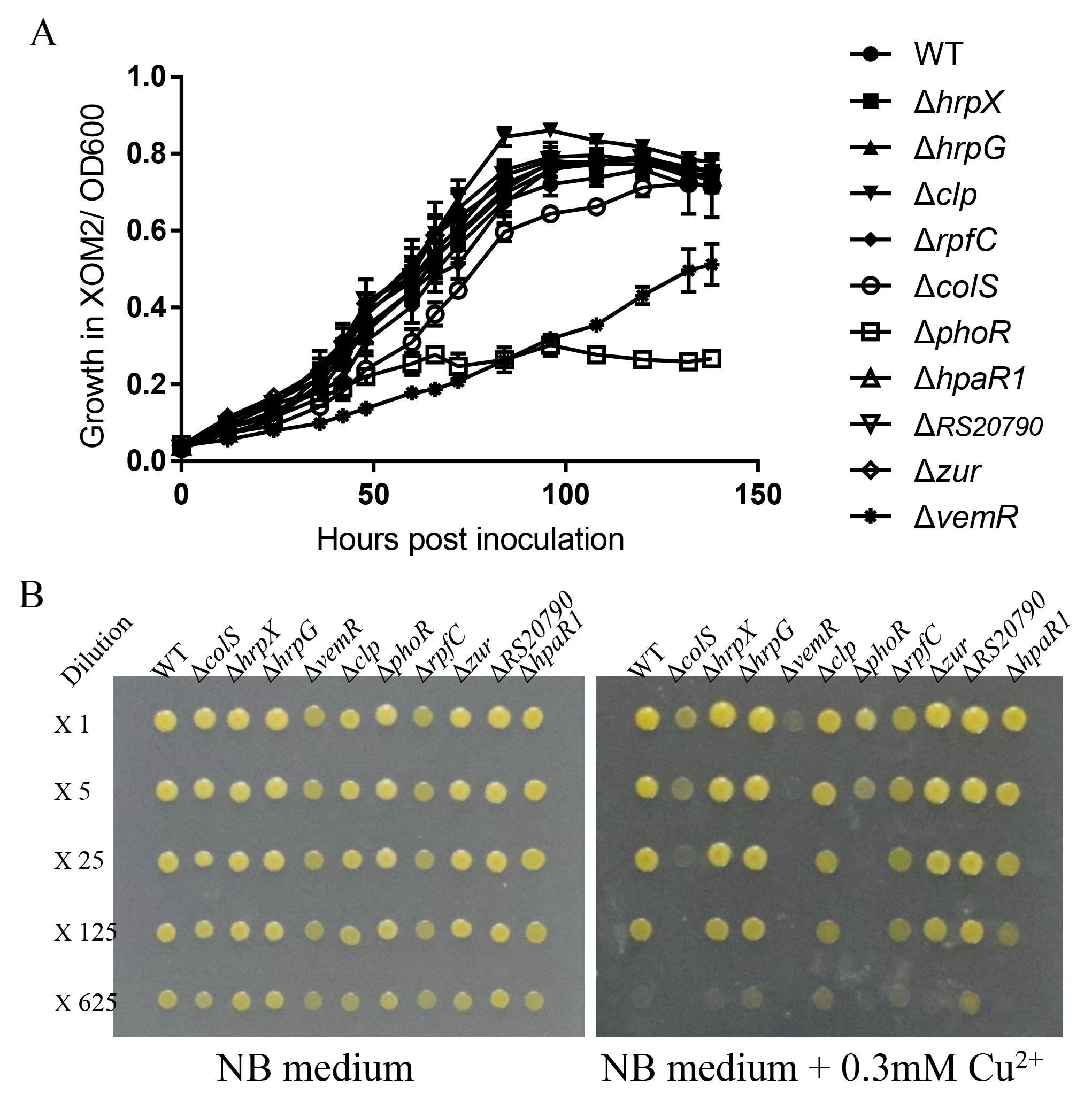

Supplement: FIG S3 [file msystems.00789-20-sf003.tif]

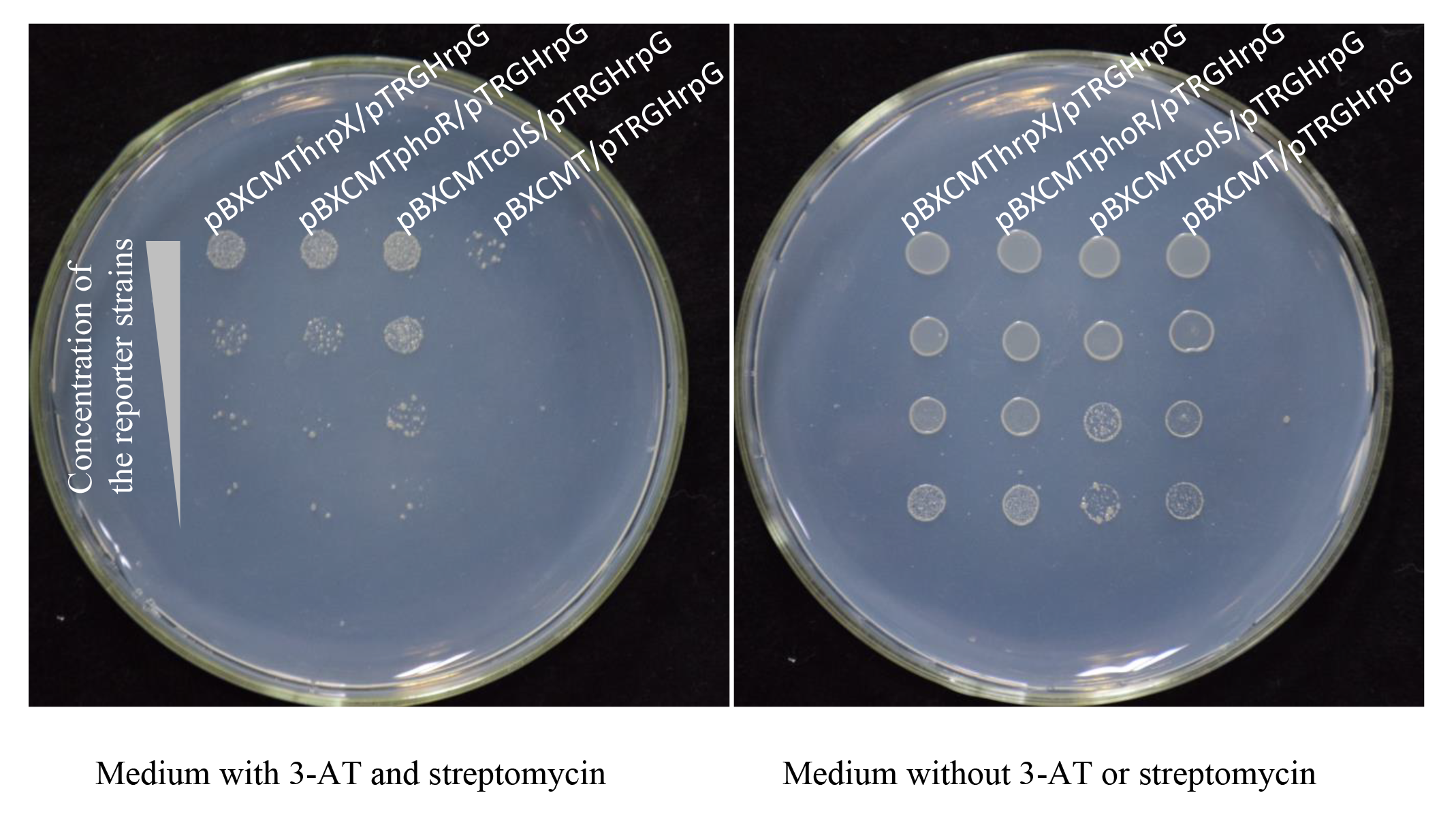

Supplement: FIG S4 [file msystems.00789-20-sf004.tif]

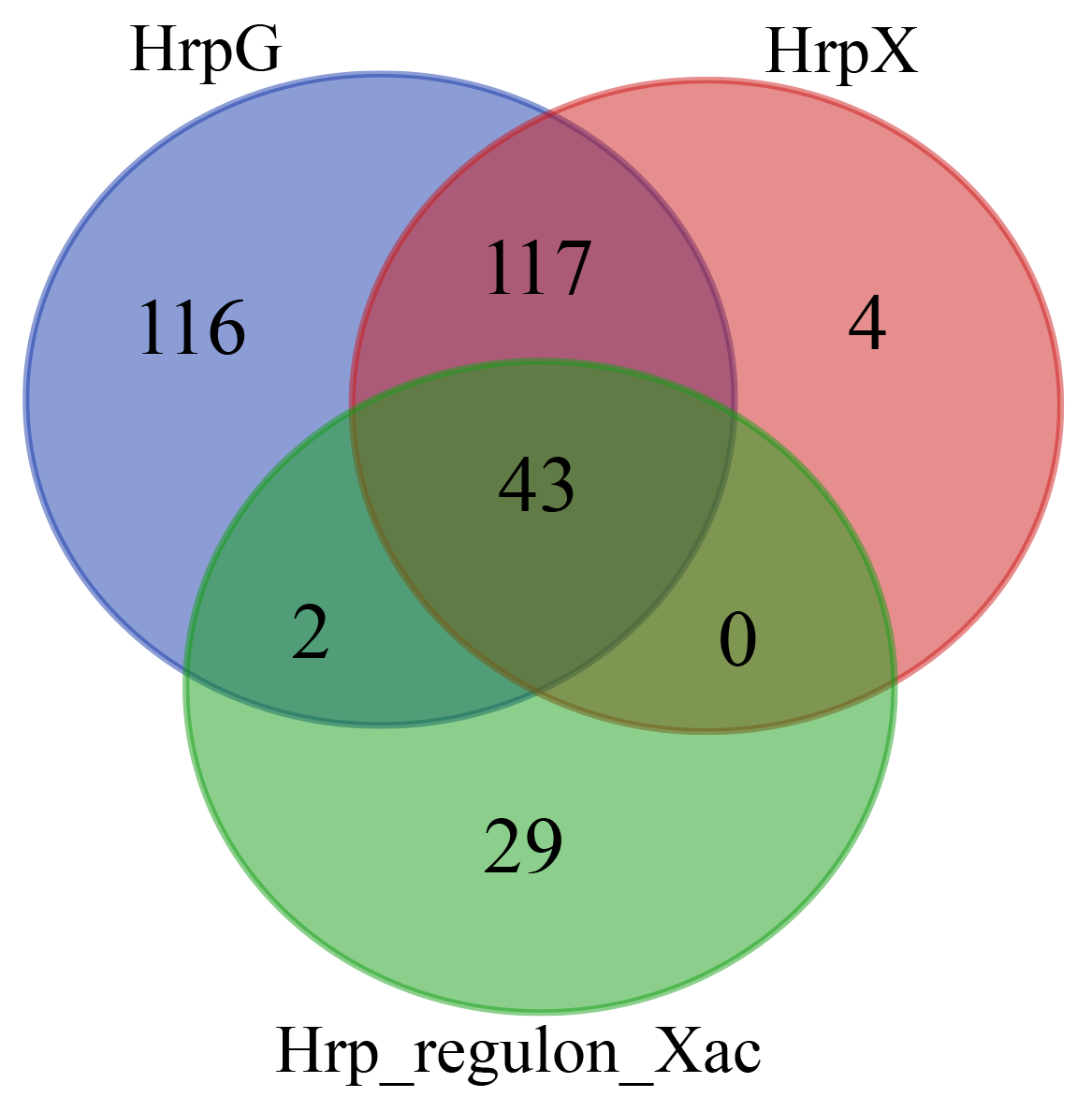

Supplement: FIG S5 [file msystems.00789-20-sf005.tif]
